# KurdABSA: Kurdish aspect-based sentiment analysis dataset curation using few-shot learning

**DOI:** 10.1016/j.dib.2025.112012

**Published:** 2025-08-29

**Authors:** Rania Azad M. San Ahmed, Soran AB. Saeed

**Affiliations:** Technical College of Informatics, Sulaimani Polytechnic University, Iraq

**Keywords:** Natural language processing, Sentiment analysis, Aspect-based sentiment analysis, Meta-learning, Low-resource, Kurdish language, Sorani dialect

## Abstract

Aspect-Based Sentiment Analysis (ABSA) extends traditional sentiment analysis by not only identifying the overall sentiment of a text but also associating specific sentiments with deeper and granular insights. The main objective of ABSA is to accurately extract relevant aspects and determine the sentiment polarity associated with each. Although extensive research has been conducted on ABSA across various languages, low-resource languages such as Kurdish remain largely underexplored in this domain. To address this gap, the present study introduces the first publicly available aspect-based sentiment analysis dataset for the Sorani dialect of Kurdish, addressing a critical gap in natural language processing (NLP) research for low-resource languages. The dataset has >4000 quadruplet ABSA in the restaurant review domain, written in the Kurdish language (Sorani dialect) using the Perso-Arabic script. A prompt-based few-shot learning model was employed to automatically annotate the dataset with aspect-opinion-category-sentiment quadruples, guided by a manually annotated support set verified by native Kurdish-language experts. This resource is intended for use in machine learning, deep learning, and cross-lingual model adaptation, making it suitable for training, fine-tuning, and benchmarking.

Specifications Table

**Table:** 

Subject	Computer Science
Specific subject area	Natural language processing, large language models, and sentiment analysis.
Type of data	Table, text.
Data collection	The data were collected from random public Facebook and Instagram pages using the Facepager tool and Python related to restaurant reviews. The basic columns in the dataset were ID, review text, aspect term, category, opinion term, and sentiment polarity. The dataset labelling has been done automatically using the power of few-shot technique model and prompt based to extract the terms efficiently
Data source location	Institution: Sulaimani Polytechnic University, Iraq. City/Country: Sulaimani, Iraq.
Data accessibility	Repository name: Mendeley Data. Data identification number: 10.17632/h5t7p4bcj2.1 Direct URL to data: https://data.mendeley.com/datasets/h5t7p4bcj2/1 Instructions for accessing these data: The dataset is public accessible following the above link
Related research article	None

## 1 Value of the Data

• The dataset, “KurdABSA,” provides the first of its kind of resources for researchers working with the Kurdish language and aspect-based sentiment analysis (ABSA). Currently, no publicly or privately available datasets exist for Aspect-based Sentiment Analysis (ABSA) in the Kurdish language for all dialects.
• KurdABSA can be widely used for various NLP tasks, such as sentiment analysis and ABSA subtasks, such as Aspect Term Extraction, Aspect Category Detection, Aspect-Opinion Pair Extraction, aspect-category-opinion-sentiment quadruple extraction, and recommendation systems.
• The labeled dataset offers a practical benchmark for evaluating the model performance. The dataset can be reused to test domain adaptation, transfer learning, or fine-tune multilingual models, making it valuable for cross-lingual evaluation.
• The KurdABSA dataset was automatically labelled using the proposed “few-shot prompt-based” methodology to reduce time and labor costs.

## 2 Background

Sentiment analysis (SA), known as “opinion mining” [1], is a subfield of Natural Language Processing (NLP) concerned with extracting subjective information from textual data to determine the emotional tone [2] and the sentiment polarity [3].

On the other hand, aspect-based sentiment analysis provides more fine-grained insights by not only determining the overall sentiment of a text but also identifying the specific aspects being discussed and the sentiment expressed toward each [4]. The ABSA framework typically comprises four core components: (1) aspect terms (A), which denote the entity or its attributes being evaluated; (2) aspect categories (C), representing predefined domain-specific labels (e.g., food, service); (3) opinion terms (O), which are subjective expressions directed at the aspects; and (4) sentiment polarity (S), indicating the sentiment orientation (positive or negative). While aspect and opinion terms are usually explicit textual spans, aspect categories and sentiment polarities are often selected from predefined class labels, collectively known as ABSA quads or quadruplets [5,6]. Recent advancements in ABSA have shifted from single-element extraction to multi-element extraction, enabling a more comprehensive analysis [7].

The Kurdish language is a member of the Indo-Iranian branch of the Indo-European language family and shares close linguistic similarities with Persian and Arabic. It is spoken by an estimated 30 to 40 million people across Iraq, Iran, Turkey, Armenia, and Syria [8]. The language is characterized by a diverse dialectal landscape, with Kurmanji (Northern Kurdish) and Sorani (Central Kurdish) being the two most widely spoken varieties. Sorani, in particular, employs the Perso-Arabic script, consisting of 36 characters (33 consonants and 3 vowels), and is written from right to left [9]. Kurdish remains underrepresented in both academic research and technological development compared to more widely studied languages.

The increasing use of the Sorani dialect on social media has led to a growing interest in NLP tasks such as text classification [9], text translation [10], idiom detection [11], stance detection [12], emotion detection [13], and sentiment analysis [8,14,15]. However, existing research remains limited to basic polarity classification, with no attention given to ABSA due to the absence of structured datasets or annotated corpora designed specifically for ABSA tasks [15].

Few-shot learning, by definition, is a machine learning approach that enables models to quickly adapt to new tasks using only a small number of labeled examples, commonly referred to as a support set [16]. One widely used implementation of this approach, particularly with the join of LLM is auto-labeling datasets with the help of a few annotated examples. The model uses these examples as contextual guidance to perform the task, without requiring additional fine-tuning [17].

Therefore, this data article presents the first of its kind of ABSA dataset in the Kurdish language. The dataset domain is related to restaurant reviews collected from social media and automatically annotated using few-shot and LLM. The dataset was verified by Kurdish language experts to ensure its accuracy. This data article supports a larger research project on sentiment analysis in Kurdish and provides a reusable resource for future studies on Sorani NLP for different tasks.

## 3 Data Description

The dataset comprises two files that are publicly accessible via the Mendeley Data repository. The files associated with this Data in Brief article are organized as follows:

### 3.1 Golden set (Few-shot_examples.csv)

This file includes 35 manually annotated examples used to guide the few-shot model. Two native speakers selected a subset of reviews and annotated them following the annotation guideline developed in section 3 to extract aspect, opinion, category, and sentiment labels. The examples are balanced for each class and include multiple quads for some of the reviews.

### 3.2 Labelled dataset (KurdABSA.csv)

This file includes **4355 records** resulting from the few-shot labelling process described in section three. Each entry in the dataset contains at least one or more ABSA quads, which include aspect terms, opinion terms, sentiment polarity, and predefined categories. Table 1 summarizes this dataset's schema.

**Table 1:** Schema of the structure of the dataset.

Column Name	Description
ID	Unique identifier for each review
Review	Full text of the customer review
Aspect Term	The specific word or phrase in the review expressing an aspect
Category Term	One of the four predefined categories (Food, Service, Environment, Price, General)
Sentiment Polarity	Sentiment polarity associated with the aspect (positive, Negative)
Opinion Term	Word or phrase conveying the opinion regarding the aspect

**Fig. 1:**
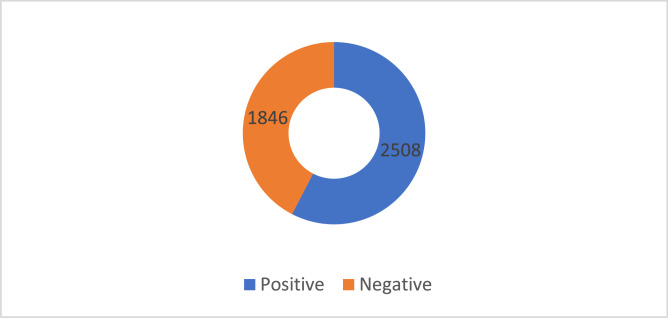
Labelled dataset distribution in terms of aspect polarity.

**Fig. 2:**
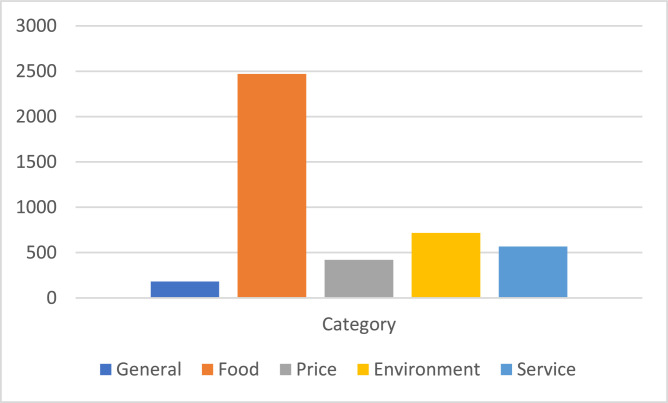
Labelled dataset distribution in terms of aspect category.

Fig. 1 represents a detailed overview of the sentiment polarity distribution within the finalized dataset, highlighting the proportion of quads labelled as positive and negative. In contrast, Fig. 2 illustrates the distribution of the aspect category classes, illustrating how frequently each predefined category appears across the dataset.

**Fig. 3:**
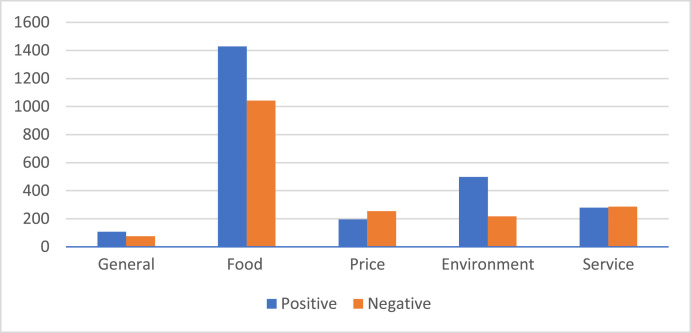
Labelled dataset distribution of polarity per aspect.

**Fig. 4:**
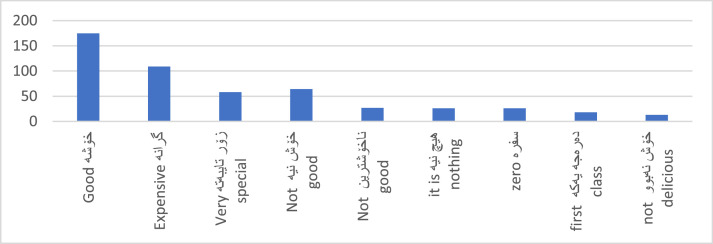
Top of opinion terms.

Fig. 3 represents the polarity distribution per aspect. Additionally, Fig. 4 provides a snapshot of the most used opinion terms by customers.

## 4 Experimental Design, Materials and Methods

**Fig. 5:**
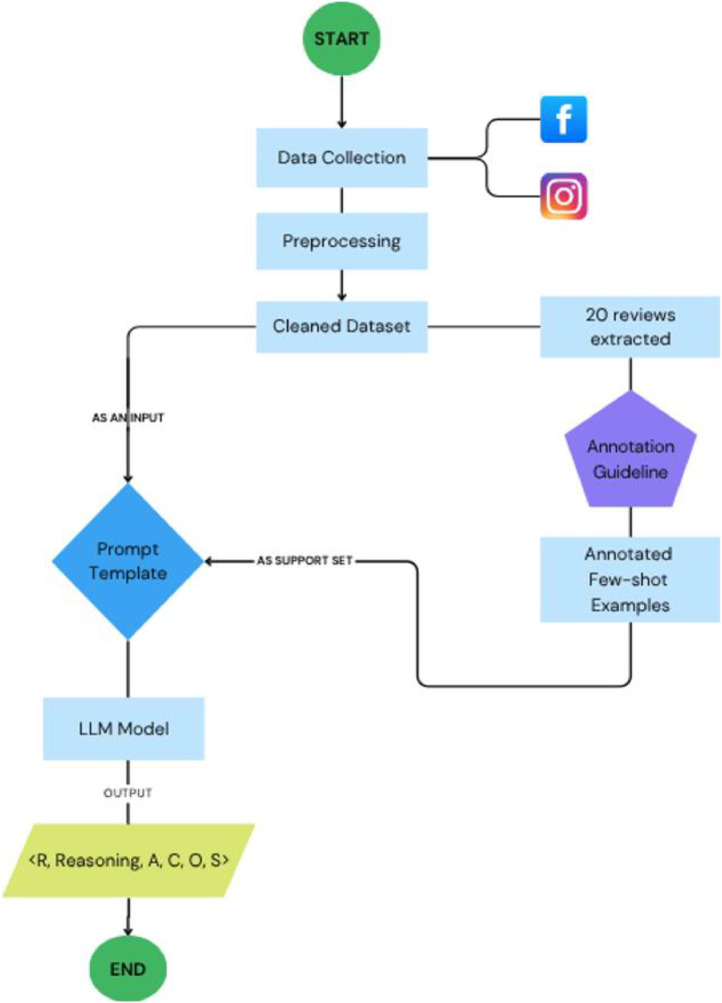
Framework of a few-shot model to auto-label the ABSA dataset.

The development of the Sorani Kurdish Aspect-Based Sentiment Analysis (ABSA) dataset was carried out through a structured multi-stage workflow, as illustrated in Fig. 5. This process began with data collection, followed by preprocessing, support set creation, and prompt template design. The dataset was then automatically annotated using a few-shot learning approach with a large language model.

### 4.1 Data collection

The dataset was collected from user comments from various public social media pages such as Facebook and Instagram, focusing on restaurant reviews within the Kurdistan Region. Given the lack of dedicated platforms for reviews—whether for books, travel, or restaurants—this task posed a notable challenge, requiring careful selection of appropriate sources. After thorough investigation, the most viable approach involved extracting user comments from relevant Facebook and Instagram pages dedicated for restaurants. The choice of this category was due that this category is reach with sentimental vocabulary. More importantly, similar datasets have been created in different languages, that is important for comparison and benchmarking later.

To facilitate this process, the Facepager tool^1^ was used to crawl publicly available content by providing the ID or link of each targeted page. All extracted comments were written in the Sorani dialect using the Perso-Arabic script. The data was then filtered to retain only review-related content, excluding very short texts, irrelevant queries (e.g., price inquiries or location), emoji-only comments, and longer responses exceeding three sentences. Additionally, all sensitive information was removed to ensure user confidentiality and uphold privacy standards.

### 4.2 Preprocessing

To ensure the reliability and consistency of the collected review data, a set of preprocessing procedures was applied as outlined in Table 2. Duplicate comments and punctuation were first removed to maintain data integrity and avoid redundancy. Emojis and non-textual symbols were also removed to minimize noise and simplify the analysis process. Finally, a language identification step was implemented to retain only those reviews written in Sorani Kurdish, excluding words containing Arabic or English. However, the misspelling correction has been intentional ignored to preserve the authenticity of the language and the characteristics of the informal writing in the social media content. LLM has the power to understand the meaning beyond the spelling mistakes using contextual embedding instead of static.

**Table 2:** Samples of before and after preprocessing.

Researchers are recommended to deal with spelling correction when they use traditional machine learning techniques.

### 4.3 Few-shot examples

Following the data collection phase, approximately 20 reviews were randomly selected to construct the few-shot examples, referred to as “Support Set”. This set served as a foundation from which the language model could learn patterns and semantic relationships during the annotation process using the LLM [18,19]. The selected set was manually annotated by two native Kurdish language experts using the annotation guideline described in section three.

To ensure class diversity and balanced representation across categories, the support set was refined to include at least two examples for each aspect category and sentiment polarity class. This balanced distribution aimed to improve the few-shot model’s ability to generalize during inference.

Furthermore, the support set incorporated both explicit and implicit aspect expressions. In cases where the aspect was implied rather than explicitly mentioned, it was annotated as NULL to maintain annotation consistency. (Table 3)

**Table 3:** Sample of few-shot examples (support set).

### 4.4 Data annotation

A comprehensive set of annotation guidelines was developed to standardize the labelling process for both the support set and subsequent dataset verification using inter-annotator agreement (IAA).

Initially, two annotators independently labeled each review. In cases of disagreement, the third expert adjudicated and provided the final annotation. It is important to note that many reviews contained multiple aspects within a single sentence. Annotators were instructed to identify and extract all valid aspect-based sentiment quads.

The refined annotation guideline is represented below; Table 4 provides the guideline of how to choose the aspect category during the annotation.

• Aspect Terms (A): These denote particular attributes or features of the entity or subject being examined. They may be explicitly stated or implied within the text and are often linked to specific categories or thematic domains.
• Opinion Term (O): These express evaluative judgments that can be either direct or subtle. Explicit opinion terms include clear indicators of sentiment—such as adjectives, adverbs, or verbs—that convey positive, negative, or neutral attitudes toward an aspect or category. Implicit opinion terms, on the other hand, suggest sentiment through more nuanced language, including humor, irony, or slang, which may allow for varied interpretations.
• Aspect Categories (C): These represent the general domain or thematic group to which a specific aspect belongs. Categories may be predefined or derived from the text and serve to contextualize sentiment analysis results (General, Food, Service, Environment, Price).
• Sentiment Polarity (S): This indicates the orientation of sentiment directed at a given aspect or category, typically classified as positive, negative, or neutral.

**Table 4:** Guide of how to choose aspect category.

Aspect Category	Topics
Food	Temperature of food, portions, menu clarity, availability of dishes, range of dishes, food taste, and dish appearance.
Service	Service speed, respect, hospitality of visitors, dealing with unexpected situations, opening time
Environment	Weather, attractive decor, comfortable seats and tables, parking availability, cleanliness of the dining area, restroom hygiene, waiting time to be seated, queue management, reservation handling, and waiting during peak hours.
Price	Customer satisfaction with prices, prices have changed from a previous visit, availability of payment methods, clarity of bill, billing errors, online payment, discounts, promotions, and loyalty programs.
General	General impressions of the restaurant, overall satisfaction, likelihood of return, comparison with previous visits, brand perception, Restaurant recommendation, perceived quality vs. expectation

### 4.5 Model implementation

Algorithm 1 outlines a prompt-based few-shot learning approach using GPT-4 Turbo to automatically extract quadruples in the format 〈Aspect, Category, Opinion, Sentiment〉 or 〈A, C, O, S〉 from each review. The process requires three main inputs: a clean dataset, a support set, and a large language model. An initially labelled dataset was created to store the results. The prompt template is then constructed using a structured, step-by-step reasoning format, which means that the template begins by defining the model’s role and task objective, followed by detailed instructions on how to extract the ABSA quadruples. Then, the support set (few-shot examples) is embedded into this prompt to guide the model in order to learn the patterns of the aspect and category of the review.

**Table:** **Algorithm 1:** Pseudocode of Few-shot using prompt-based GPT-4 Turbo.

1: **Require:** Clean Dataset (R), Support_Set (S), Language_Model (*M* = GPT-4 Turbo) 2: Initialize Labelled_Dataset (D) ← ∅ 3: **Load** R and S 4: Create Prompt_Template (P) using step-by-step reasoning structure 6: Embed S into P → P_base 7: **for** each review r ∈ Preprocessed_Reviews do 8: Prompt ← P_base + ``Review: `` + *r* + '' Extract Quads:'' 9: Output ← Call_Model (M, Prompt) 10: Reasoning ← Extract intermediate reasoning steps from Output 11: (A, C, O, S) ← Extract ABSA Quads from Reasoning 12: Append (r, Reasoning, A, C, O, S) to D 13: **end for** 14: **return** D

This prompt was passed to the GPT-4 Turbo model, the output was extracted from the reasoning output, and the corresponding ABSA quads were derived. These components, along with the original review and reasoning traces, were appended to the labeled dataset. After iterating through all reviews, the algorithm returned a complete annotated dataset. This method leverages GPT-4′s reasoning capabilities and language understanding to perform ABSA in a few-shot setting without requiring fine-tuning.

**Fig. 6:**
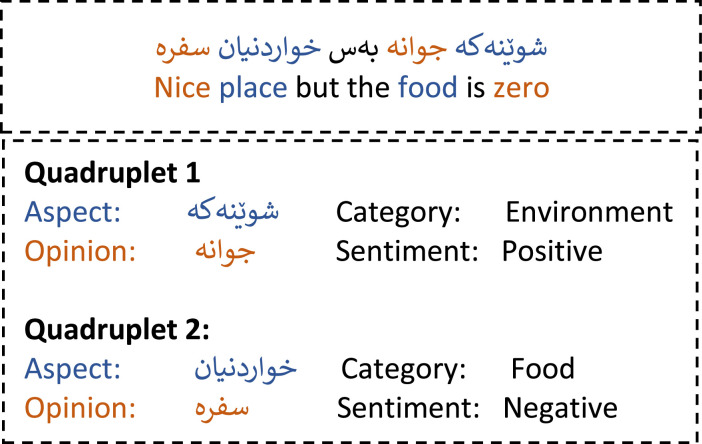
Sample of the output of the few-shot model.

Fig. 6 represents the quadruplet outcome of one review. The sentence contains two quadruplets, extracted by the model.

### 4.6 Inter-Annotator agreement

To verify the reliability and consistency of the dataset, the same annotators verified random samples, assessing both category and polarity annotations based on the proposed guideline. During the verification process, any disagreements were recorded and reviewed. Both annotators identified the same instances of disagreement, eliminating the need for a third annotator. Cohen’s kappa coefficient (κ) was calculated to evaluate the level of agreement between the annotators and was found to be 0.87. Two rounds of verification were conducted with random samples. All conflicts were resolved and corrected in the dataset to ensure its consistency, and reliability for future use.

## Limitation

Several limitations were encountered during dataset development. First, the dataset size is relatively limited because most of the reviews are written either in Latin script or in English.

On the other side, the dataset is domain-specific, as it focuses exclusively on restaurant reviews. Consequently, the language and sentiment expressions captured may not be fully generalizable to other domains, such as politics, education, and healthcare.

Finally, owing to the informal nature of social media texts, challenges such as spelling variations, use of non-standard orthography, and mixed language code-switching pose difficulties during data preprocessing and may affect model performance in downstream tasks if not addressed carefully.

## Ethics Statement

The authors have read and followed the ethical requirements for publication in Data in Brief and confirm that the current work does not involve human subjects or animal experiments. The data collected was publicly available and completely anonymous, no personal or sensitive information is included, and there are no ethical concerns regarding privacy or data usage associated with this study.

## Credit Author Statement

**Rania Azad M. San Ahmed:** Data curation, methodology, visualization, investigation, writing, and original draft preparation. **Soran AB. Saeed:** Supervision, Writing, Reviewing, and Editing.

## Data Availability

Mendeley DataKurdABSA (Original data).

## Footnotes

1 https://github.com/strohne/Facepager.
